# Assessment of cognitive biases and biostatistics knowledge of medical residents: a multicenter, cross-sectional questionnaire study

**DOI:** 10.3402/meo.v19.23646

**Published:** 2014-03-12

**Authors:** Pavlos Msaouel, Theocharis Kappos, Athanasios Tasoulis, Alexandros P. Apostolopoulos, Ioannis Lekkas, Elli-Sophia Tripodaki, Nikolaos C. Keramaris

**Affiliations:** 1 Greek Junior Doctors and Health Scientists Society, Athens, Greece; 2 2nd Department of Surgery, Metaxa Cancer Memorial Hospital, Pireaus, Greece; 3 Department of Clinical Therapeutics, Alexandra Hospital, Athens, Greece; 4 Department of Orthopaedics, Asklipiio Hospital, Athens, Greece; 5 Otorhinolaryngology Clinic, 251 G.N.A. Hospital, Athens, Greece; 6 1st Department of Internal Medicine, Evangelismos Hospital, Athens, Greece

**Keywords:** biostatistics knowledge, cognitive bias, conjunction fallacy, gambler’s fallacy, medical residents, statistical literacy

## Abstract

**Purpose:**

The aim of this study is to determine the perceived familiarity of medical residents with statistical concepts, assess their ability to integrate these concepts in clinical scenarios, and investigate their susceptibility to the gambler’s fallacy and the conjunction fallacy.

**Methods:**

A multi-institutional, cross-sectional survey of Greek medical residents was performed. Participants were asked to indicate their familiarity with basic statistical concepts and answer clinically oriented questions designed to assess their biostatistics knowledge and cognitive biases. Univariate, bivariate, and multivariate statistical models were used for the evaluation of data.

**Results:**

Out of 153 respondents (76.5% response rate), only two participants (1.3%) were able to answer all seven biostatistics knowledge questions correctly while 29 residents (19%) gave incorrect answers to all questions. The proportion of correct answers to each biostatistics knowledge question ranged from 15 to 51.6%. Residents with greater self-reported familiarity were more likely to perform better on the respective knowledge question (all *p*<0.01). Multivariate analysis of the effect of individual resident characteristics on questionnaire performance showed that previous education outside Greece, primarily during medical school, was associated with lower biostatistics knowledge scores (*p<*0.001). A little more than half of the respondents (54.2%) answered the gambler’s fallacy quiz correctly. Residents with higher performance on the biostatistics knowledge questions were less prone to the gambler’s fallacy (odds ratio 1.38, 95% confidence intervals 1.12–1.70, *p=*0.003). Only 48 residents (31.4%) did not violate the conjunction rule.

**Conclusions:**

A large number of medical residents are unable to correctly interpret crucial statistical concepts that are commonly found in the medical literature. They are also especially prone to the gambler’s fallacy bias, which may undermine clinical judgment and medical decision making. Formalized systematic teaching of biostatistics during residency will be required to de-bias residents and ensure that they are proficient in understanding and communicating statistical information.

## Introduction

Biostatistical literacy is imperative for physicians in training who must keep abreast of current medical knowledge and be able to communicate statistical and epidemiological information to patients and colleagues. Furthermore, medical residents may be particularly vulnerable to the suggestions of the published literature and other more questionable sources such as promotional brochures and websites (1, 2). The vast majority of published medical research contains at least some elementary statistical analysis, and it has consistently been shown that errors in such data are frequently found, even in well-respected textbooks (3–7). It is therefore crucial for residents to possess the necessary basic biostatistical knowledge to critically evaluate and apply original research data.

Previous surveys conducted in the 1980s and early 1990s demonstrated that physicians have poor understanding of simple biostatistical concepts (8–11). Physicians today may have even more pronounced difficulties in comprehending and integrating data derived from the increasingly sophisticated statistical methodologies used in contemporary medical literature (7, 12–14). More recent studies have shown that internal medicine, emergency medicine, and obstetrics–gynecology residents have considerable difficulties in interpreting statistical results found in published clinical research (15–17). Therefore, despite the fact that many medical schools include statistical courses in their curricula, it should not be taken for granted that residents can effectively assess biostatistical information and use it to the best advantage of their patients.

A number of predictable biases in human information processing may considerably hinder sound clinical decision making (18–21). Therefore, documenting and elucidating these processes may improve patient outcomes, prevent the inappropriate utilization of medical resources, and reduce health care expenditures. A previous instrument designed to evaluate such cognitive biases among physicians (22) was recently reported to have very limited measurement properties (23). The representativeness heuristic is a known factor that may lead to bias in probability estimation by physicians (24, 25). The gambler’s fallacy is a classic cognitive bias produced by the representativeness heuristic and arises when a person assumes that a deviation from what occurs on average or in the long term will be corrected in the short term (26). A common example is when tails are repeatedly obtained while tossing a fair coin leading the gambler to incorrectly expect that heads are ‘due’ for the next toss. In reality, the previous tosses have no bearing on the outcome of future tosses. The gambler’s fallacy has been shown to be by far the most frequent bias arising in this probability judgment situation, particularly among highly educated individuals (27, 28). The conjunction fallacy is another cognitive illusion of probability judgment which was first documented using the well-known ‘Linda’ example whereby the stereotypical description of Linda as a social activist compels respondents to rank the probability that she is a feminist bank teller as greater than the probability that she is a bank teller. However, this response is considered erroneous as feminist bank tellers cannot be more frequent or more probable than the more inclusive class of bank tellers (29).

The aim of the present multicenter, cross-sectional study was to determine the perceived familiarity of medical residents with statistical concepts and assess their ability to integrate these concepts in actual clinical scenarios. Furthermore, the incidence of the gambler’s fallacy and the conjunction fallacy among medical residents were investigated using clinical vignettes that were designed to tap these two domains with specific reference to medicine. The effects of individual resident characteristics such as age, gender, residency year, and past biostatistics training on the cognitive biases and biostatistics knowledge of medical residents were also evaluated.

## Methods

### Participants and data collection

The reporting of this cross-sectional study conforms to the STROBE statement (30). The data presented here are part of a large multi-institutional project evaluating the biostatistical competency and cognitive biases of medical residents. Respondents were originally recruited to participate in a cross-sectional randomized experimental study of medical resident’s Bayesian reasoning performance and the sample size was determined according to that study’s requirements. The survey was conducted from January to March 2010, and the participants were chosen from eight major Greek hospitals in the city of Athens (Table 1). The Greek system does not allow official residency training in private hospitals and therefore all institutions surveyed in this study were public. Every resident was given a number according to the official enrolment lists that had been provided by each of the hospitals. Out of 1,272 eligible residents in all hospitals, 200 (25 from each hospital) were randomly selected by a computerized method to participate in the sample, a number that represents approximately 2% of the total number of residents training in Greece (31). Participation was elective and all participants were approached during breaks from work or training, were informed that responses would be anonymous and were blinded to the scope and purpose of the study. The residents were asked to return the completed questionnaires to a sealed box provided in each hospital. The study received permission from the authorized personnel at each institution (i.e., the Department or Scientific Meeting Chair). The study complied with Greek requirements for survey studies, and ethical approval was not required as responses were fully anonymous, participation was elective, all participants were approached during breaks from work or training, and the study did not contain questions on sensitive topics. Participants’ informed consent was indicated by each individual’s willingness to complete and return the questionnaire.

**Table 1:** Resident demographic, educational, and residency profile

**1. Age**	Median: 32 years Range: 24–44 years		**2. Gender** a	Male	Female	**3. Year of residency**	Median: 2 years Range: 1–7 years	
				112 (73.2%)	41 (26.8%)			

**4. Hospital** a^**,**^^b^	1st I.K.A. Hospital	251 G.N.A. Hospital	Aglaia Kyriakou Hospital	Alexandra Hospital	Asklipiio Hospital	Elpis Hospital	Evangelismos Hospital	Metaxa Hospital
	18 (11.8%)	17 (11.1%)	21 13.7%)	21 (13.7%)	21 (13.7%)	19 (12.4%)	15 (9.8%)	21 (13.7%)

**5. Residency field** ^**a**^	Medical specialties			Surgical specialties	Diagnostic and laboratory specialties			
	71 (46.4%)			67 (43.8%)	15 (9.8%)			

**6. Other graduate degrees**^a^^,^^c^	No 136 (88.9%)		PhD 6 (3.9%)	MSc 14 (9.2%)		** 7. Residency program type**^a^	University based 28 (18.3%)	Community based 125 (81.7%)

**8. Education abroad**^a^^**,**^^d^	No		Primary or secondary education	Medical School		Prior to beginning residency	During Residency	Other
	96 (62.7%)		2 (1.3%)	45 (29.4%)		4 (2.6%)	10 (6.5%)	3 (2%)

**9. Biostatistics training** a^,^^e^	No		Primary or secondary education	Medical School		Prior to beginning residency	During Residency	Other
	28 (18.3%)		7 (4.6%)	119 (77.8%)		0	6 (3.9%)	11 (7.2%)

a Percentages in parentheses represent the rate percent to the study’s 153 respondents.

b Percentages in parentheses do not add up to 100% due to rounding.

c Percentages in parentheses do not add up to 100% due to rounding and overlapping of degrees (three respondents had both PhD and MSc degrees).

d Percentages in parentheses do not add up to 100% due to rounding and overlapping of foreign educational experiences.

e Percentages in parentheses do not add up to 100% due to rounding and overlapping of past biostatistics training settings.

### Survey measures

The full questionnaire was developed in the Greek language and is available from the corresponding author. The first seven questions queried the socio-demographic profile, specialty choices, previous education outside Greece, current training level, residency year, and past training in biostatistics of residents. We combined residencies according to their conceptual and occupational relations and formed three different medical ‘fields’: 1) internal medicine (*n=*71; dermatology, pediatrics, neurology, and general practice were also included in this group); 2) surgical specialties (*n=*67); and 3) diagnostic and laboratory specialties (*n=*15). The participants were then asked to indicate their understanding of the statistical concepts of standard deviation (SD), standard error of the mean (SEM), *p*-values, confidence intervals (CI), correlation coefficients, relative risk (RR), sensitivity, and positive predictive value (PPV) by rating their familiarity with each concept on a 7-point Likert scale ranging from ‘none’ (score of 1) to ‘excellent’ (score of 7). The next set of questions was developed by one of the authors and assessed the biostatistics knowledge (Table 2) and cognitive biases (Table 3) of medical residents.

**Table 2:** Biostatistics knowledge questions

	Scenario description	Multiple choices (select only one)	Response frequencies^a^ (%)
SD vignette	You read in a medical paper that 100 patients had fasting serum glucose levels of 153 mg/dl±6 mg/dl (mean±SD). Which of the following statements is the most correct?	• It is approximately 95% certain that the true mean value lies between 147 and 159 mg/dl	80 (52.3)
		• Approximately 50% of patients had fasting serum glucose 153 mg/dl	12 (7.8)
		• Approximately 95% of patients had fasting serum glucose 141–165 mg/dl	49 (32)^b^
		• I do not know and do not wish to guess	12 (7.8)
SEM vignette	You read in a medical paper that 2 hours following administration of a drug the mean systolic pressure of the patients was 138 mm Hg±8 mm Hg (mean±SEM). Which of the following statements is the most correct?	• It is approximately 95% certain that the true mean value lies between 122 and 154 mm Hg	35 (22.9)^b^
		• Approximately 50% of patients had mean systolic pressure 138 mm Hg	11 (7.2)
		• Approximately 95% of patients had mean systolic pressure 130–146 mm Hg	85 (55.6)
		• I do not know and do not wish to guess	22 (14.4)
*p*-value vignette	You read in a clinical trial report that the treatment under investigation showed statistically significant increase in overall survival compared to the placebo-control group (*p<*0.05). Which of the following statements is most correct?	• The treatment is definitely better compared to placebo	58 (37.9)
		• The observed increase in overall survival is so large that there is less than a 5% chance that placebo is equal to the treatment	30 (19.6)
		• If the treatment does not actually increase survival, then the chance of obtaining the observed (or even greater) increase in survival is less than 5%.	41 (26.8)^b^
		• I do not know and do not wish to guess	24 (15.7)
CI vignette	You read in a medical paper that the RR for a disease in individuals who are exposed to a risk factor is 1.64 (95% CI=1.10–2.45) compared to otherwise similar individuals who are not exposed to this risk factor. Which of the following statements is most correct?	• 95% of those exposed to the risk factor had RR between 1.10 and 2.45	44 (28.8)
		• There is a 95% likelihood that the true RR falls between 1.10 and 2.45	51 (33.3)^b^
		• Approximately 50% of individuals exposed to the risk factor had a RR of 1.64	9 (5.9)
		• I do not know and do not wish to guess	49 (32)
Correlation coefficient vignette	You read in a medical paper that there was a highly statistically significant positive correlation between atrial natriuretic peptide blood levels and treatment outcomes (*r=*0.29, *p<*0.001). Which of the following statements is most correct?	• There was a strong correlation between atrial natriuretic peptide blood levels and treatment outcomes	97 (63.4)
		• The correlation between atrial natriuretic peptide blood levels and treatment outcomes was weak	23 (15)^b^
		• No correlation between atrial natriuretic peptide blood levels and treatment outcomes was detected	4 (2.6)
		• I do not know and do not wish to guess	29 (19)
Sensitivity vignette	An article is describing the effectiveness of a novel diagnostic technique for the detection of a disease. The sensitivity of the technique is defined as:	• The ratio of true positives to the number of subjects who have the disease	79 (51.6)^b^
		• The ratio of true positives to the number of subjects who had positive test results	54 (35.3)
		• The ratio of false positives to the number of subjects who had positive test results	8 (5.2)
		• I do not know and do not wish to guess	12 (7.8)
RR vignette	You read in a medical paper that a new screening test for a disease reduces by 25% the risk of death from the disease in high-risk individuals compared to those who do not undergo the screening test (RR=0.75). On the other hand, you are also aware that 12 out of 1,000 high-risk individuals who undergo an older screening test will die from the disease compared to 20 out of 1,000 high-risk individuals who are not screened with any method. Which of the following statements is most correct?	• The data indicate that the new screening test may achieve greater reduction of the risk of death from the disease compared to the old screening test	49 (32)
		• The data indicate that the old screening test may achieve greater reduction of the risk of death from the disease compared to the new screening test	43 (28.1)^b^
		• The data indicate that both screening tests have similar efficacy in reducing the risk of death from the disease	10 (6.5)
		• I do not know and do not wish to guess	51 (33.3)

a Percentages in parentheses represent the rate percent to the study’s 153 respondents.

b Correct answer. SD, standard deviation; SEM, standard error of the mean; CI, confidence interval; RR, relative risk.

**Table 3:** Cognitive biases questions

	Scenario description	Multiple choices (select only one)	Response frequencies^a^ (%)
Gambler’s fallacy vignette	Approximately 10 patients with hemoptysis per month attend a primary care center. Epidemiological data from this center indicate that approximately 1 out of 10 hemoptysis patients will have TB. Near the end of the month, nine patients with hemoptysis have attended the center and all were TB-negative. Which of the following statements about the next patient with hemoptysis who will attend the primary center before the end of the month is most correct?	• It is almost 100% likely that the patient will have TB	4 (2.6)
		• The patient’s chances of having TB will be approximately 50%	7 (4.6)
		• The patient’s chances of having TB will be approximately 10%	83 (54.2)^b^
		• The patient’s likelihood of having TB will be increased compared to the previous 9 patients with hemoptysis but more data will be required to calculate it	30 (19.6)
		• No conclusions about the patient can be derived from the scenario’s data	29 (19)
Conjunction fallacy vignette	John is a 42 years old, married office worker with a BMI of 35.6. He complains of angina-like chest pain. His father had CAD and died from myocardial infarction 7 years ago. John’s brother also suffers from CAD. Based on the above data, rank the following statements from one to six where one is the most probable and six is the least probable statement:	• John has depression	48 (31.4) respondents ranked the likelihood that John has Huntington’s disease higher compared to the likelihood that he has both CAD and Huntington’s disease
		• John is suffering from osteosarcoma and pneumonia	
		• John has CAD	
		• John has Huntington’s disease	
		• John has pre-diabetes	
		• John has CAD and Huntington’s disease	

a Percentages in parentheses represent the rate percent to the study’s 153 respondents.

b Correct answer. BMI, body mass index; TB, tuberculosis; CAD, coronary artery disease.

A mathematician from the Section of Statistics and Operations Research of the Department of Mathematics of the University of Athens reviewed the questions for clarity and mathematical appropriateness. In order to assess the intelligibility, interpretation, content validity, adequacy of response options, and clinical relevance of the vignettes, the questionnaire was pretested in a separate group of seven medical residents, including two residents with advanced training in epidemiology and biostatistics. The residents completed the questionnaire and provided oral feedback which resulted in the removal of one question to avoid duplication of similar concepts. Both residents with advanced biostatistics backgrounds answered all questions correctly. Of the remaining five residents, four answered four of seven biostatistics knowledge questions correctly while one resident correctly answered two out of seven questions. Four of these five residents selected the ‘approximately 10%’ choice in the gambler’s fallacy scenario and answered that the probability of suffering from coronary artery disease (CAD) and Huntington’s disease is greater than the probability of suffering from Huntington’s disease. Despite extensive discussion of the conjunction fallacy vignette’s probabilistic nature, these residents were very reluctant to accept the strictly mathematical reading of the problem.

A ‘total biostatistics knowledge’ score, which equally weighted each of the seven questions listed in Table 2, was calculated. Each correct response counted one point while no points were given for incorrect answers. Furthermore, a ‘total biostatistics familiarity’ score was calculated by adding all familiarity ratings.

### Statistical analysis

Data analysis was performed using R (Foundation for Statistical Computing, Vienna, Austria) (32). Variables were maintained as continuous or categorical according to their original form in the questionnaire. A non-normal distribution was assumed for all continuous variables as indicated by the Kolmogorov–Smirnov test. Missing values were counted as incorrect responses and added in the ‘I do not know and do not wish to guess’ response selection (Table 2). Median differences between more than two groups were evaluated using the Kruskal–Wallis one-way analysis of variance by ranks. The Spearman rank correlation coefficient was used to determine the correlation between two continuous variables. Median differences between two groups were analyzed using the Mann–Whitney U test for two non-paired data. Categorical variables were compared using Pearson’s Chi-square or Fisher’s exact tests where appropriate. The internal consistency of the ‘total biostatistics familiarity’ and the ‘total biostatistics knowledge’ scores was assessed by examining the Cronbach’s α coefficient. Cronbach’s α>0.6 were considered to be acceptable (33). Bivariate analyses were performed to identify individual resident characteristics that may be associated with total biostatistics knowledge score, total biostatistics familiarity score, or performance on the cognitive biases questions. Candidate factors included age, gender, hospital, residency field, residency year, residency program type, possession of other advanced degrees, education abroad, past biostatistics training, and total biostatistics familiarity score. Variables with *p*<0.05 on bivariate comparisons were further included in multivariate regression analyses. Multivariate analysis was performed with linear regression analyses as well as binary logistic regression tests using the dichotomous coding of responses (correct/incorrect) as the dependent variable. To adjust for multiple pairwise comparisons, *p*<0.01 was considered statistically significant.

## Results

The survey response rate was 76.5% (153/200). Table 1 details participant demographic profile, education, and specialty choice. The majority of residents (81.7%) reported prior attendance of biostatistics courses, which mainly occurred during medical school.

Responses to each of the individual biostatistics knowledge and cognitive bias questions are presented in Tables 2 and 3, respectively. The proportion of correct answers to each biostatistic knowledge question ranged from 15% in the scenario concerning correlation coefficients to 51.6% in the diagnostic sensitivity question. The most popular incorrect answer (selected by 63.4% of respondents) in the correlation coefficient question stated that the correlation between the two variables was strong. However, the correlation coefficient provided in the vignette (*r=*0.29) was actually low. Only two participants (1.3%) answered all biostatistics knowledge questions correctly while 29 residents (19%) gave incorrect answers to all seven questions. Reliability analysis of the ‘total biostatistics familiarity’ and the ‘total biostatistics knowledge’ scores showed acceptable α values of 0.904 and 0.648, respectively. The mean±SD of the ‘total biostatistics knowledge’ score was 2.1±1.8 (range 0–7). No significant association was found between residency field and total biostatistics knowledge score (*p=*0.066). On the other hand, bivariate analyses indicated a significant association of total biostatistics knowledge score with residents’ age (*r=*−0.166, *p=*0.041), training year (*r=*−0.205, *p=*0.011), total biostatistics familiarity score (*r=*0.333, *p<*0.001), and education abroad (*p=*0.004). Multivariate analysis was further performed in order to estimate the independent effects of these factors on biostatistics knowledge (Table 4). Residents’ perceived familiarity with biostatistics was associated with higher total knowledge scores (*p<*0.001) while previous education outside Greece was associated with lower total knowledge scores (*p=*0.005).

**Table 4:** Multivariate association (multiple linear regression) between resident variables and total biostatistics knowledge score

	Regression coefficient^a^	*p*
Age (years)	0.046 (−0.059 to 0.150)	0.389
Year of residency	−0.227 (−0.429 to −0.026)	0.027
Education abroad^b^	−0.846 (−1.426 to −0.266)	0.005^c^
Total biostatistics familiarity score	0.047 (0.026 to 0.068)	<0.001^c^

a Numbers in parentheses represent the 95% confidence intervals (CI).

b ‘No education abroad’ is used as the reference category.

c Statistically significant association set at *p<*0.01 to adjust for multiple pairwise comparisons.

Perceived familiarity ratings of biostatistical concepts by medical residents are listed in Table 5. Thirty-one residents (20.3%) gave scores ≤4 on all familiarity ratings. Notably, <25% of residents reported having above average knowledge (familiarity rating>4) of correlation coefficients. The mean familiarity score for this statistical concept was 2.9±1.9 (mean±SD) which was significantly lower (*p<*0.01) compared to all other familiarity ratings. Bivariate analyses showed that residents with higher self-reported familiarity with SD (*p<*0.001), SEM (*p=*0.001), *p*-values (*p=*0.009), CI (*P<*0.001), and correlation coefficients (*p<*0.001) performed better on the respective knowledge question. Also, there was no significant association between self-reported understanding of the concepts of RR (*p=*0.134) and sensitivity (*p=*0.571) with the respective knowledge question. The mean±SD of the ‘total biostatistics familiarity’ score was 31.6±12.4 (range 8–55). In bivariate analyses, total biostatistics familiarity was significantly associated with past training in biostatistics (*p=*0.006) and possession of other advanced degrees (*p<*0.001). Conversely, residency field was not significantly associated with total biostatistics familiarity (*p=*0.236). A linear regression model was fitted with ‘total biostatistics familiarity’ score as the dependent variable and past biostatistics training and possession of other advanced degrees as the independent variables (Table 6). Residents who have previously attended biostatistics courses tended to report higher familiarity scores although this effect did not reach statistical significance (*p>*0.01). Residents with other advanced degrees were significantly more likely to report higher familiarity with statistical concepts (*p<*0.001).

**Table 5:** Perceived familiarity rating by medical residents for the biostatistical concepts of SD, SEM, p-values, CI, correlation coefficients, RR, sensitivity, and PPV

Familiarity rating^a^^,^^b^	1 (none) (%)	2 (%)	3 (%)	4 (%)	5 (%)	6 (%)	7 (excellent) (%)
SD	24 (15.7)	7 (4.6)	26 (17)	11 (7.2)	28 (18.3)	26 (17)	31 (20.3)
SEM	37 (24.2)	10 (6.5)	23 (15)	24 (15.7)	24 (15.7)	19 (12.4)	16 (10.5)
*P*	34 (22.2)	13 (8.5)	16 (10.5)	21 (13.7)	19 (12.4)	29 (19)	21 (13.7)
CI	46 (30.1)	16 (10.5	23 (15)	19 (12.4)	24 (15.7)	14 (9.2)	11 (7.2)
Correlation coefficients	54 (35.3)	21 (13.7)	30 (19.6)	14 (9.2)	11 (7.2)	13 (8.5)	10 (6.5)
RR	33 (21.6)	11 (7.2)	26 (17)	23 (15)	23 (15)	36 (23.5)	1 (0.7)
Sensitivity	14 (9.2)	7 (4.6)	14 (9.2)	16 (10.5)	14 (9.2)	36 (23.5)	52 (34)
PPV	20 (13.1)	12 (7.8)	15 (9.8)	24 (15.7)	22 (14.4)	30 (19.6)	30 (19.6)

a Percentages in parentheses represent the rate percent to the study’s 153 respondents.

b Percentages in parentheses do not add up to 100% due to rounding. SD, standard deviation; SEM, standard error of the mean; CI, confidence intervals; RR, relative risk; PPV, positive predictive value.

**Table 6:** Multivariate association (multiple linear regression) between resident variables and total biostatistics familiarity score

	Regression coefficient^a^	*p*
Biostatistics training^b^	6.054 (1.205 to 10.904)	0.015
Other graduate degrees^c^	11.349 (5.382 to 17.316)	<0.001^d^

a Numbers in parentheses represent the 95% confidence intervals (CI).

b ‘No prior biostatistics training’ is used as the reference category.

c ‘No other graduate degrees’ is used as the reference category.

d Statistically significant association set at *p<*0.01 to adjust for multiple pairwise comparisons.

As shown in Table 3, approximately half of the respondents (54.2%) answered the gambler’s fallacy quiz correctly. Furthermore, less than one third of residents surveyed (31.4%) judged the probability that John has Huntington’s disease to be greater than the probability that John has both CAD and Huntington’s disease. Preliminary bivariate analysis of factors that may affect resident performance on the gambler’s fallacy problem was followed by multivariate logistic regression which showed that residents with higher total biostatistics scores were more likely to answer that quiz correctly (odds ratio=1.38, 95% CI 1.12–1.70; *p=*0.003). Also, none of the investigated individual resident factors were found to be significantly associated with performance on the conjunction fallacy quiz.

## Discussion

The present multicenter survey of biostatistics performance and biases demonstrated that a considerable proportion of medical residents are prone to errors and biases that undermine judgement and decision making. The fact that, on average, only two out of seven biostatics knowledge questions were answered correctly raises serious concerns. This significant lack of biostatistics aptitude was observed despite the fact that the majority of participants have attended undergraduate biostatistical training during medical school. It should be noted, however, that most residents did not reinforce these courses during residency training. Indeed, the biostatistics knowledge score tended to decline with progression through the years of residency training although this observation did not reach statistical significance (Table 4). Furthermore, we did not observe a significant performance difference between residents who have previously received biostatistics education with those that have never received such training at any point in their career. Taken together, these data suggest that biostatistics skills may atrophy with progression through medical training and further effort should be expended to maintain knowledge in that sphere during residency.

The majority of residents were unable to properly combine significance level information with correlation coefficient data (Table 2). This mistake was observed even in respondents who have selected the correct definition of the *p*-value in the relevant question. Educators may therefore need to reevaluate how these concepts are taught and represented in a way that medical residents can understand and use in rational, evidence-based decisions.

Further analysis of the effects of individual resident variables (Table 4) revealed that respondents (all Greek citizens) who have received medical education outside Greece, predominantly taking place during undergraduate medical education in international medical schools, had lower biostatistics knowledge scores compared to residents who have never trained abroad. This performance difference may be partly due to variability in medical school experiences obtained by Greek citizens abroad but it may also reflect differences in ability and effort. Further research will be required to elucidate the potential performance differences between graduates of Greek and international medical schools particularly with regards to educational quality, clinical outcomes and quality of care. It should also be noted that other resident characteristics, including age, gender, advanced Graduate training, specialty choice, hospital and residency program type did not affect the biostatistics knowledge score of residents indicating that the insufficient biostatistical literacy reported in the present study is similar across a broad range of medical residents in Greece. Furthermore, biostatistical performance was homogenous among residents training in different specialty programs. To our knowledge, the present study is the first to investigate and directly compare the biostatistics knowledge between different resident specialty fields. Recent studies have assessed the biostatistical competence of internal medicine, emergency medicine, and obstetrics–gynecology residents (15–17). Contrary to the present study, Windish et al. reported that prior biostatistics training, gender and advanced degrees were predictive of internal medicine residents’ statistics knowledge (15). Also, a Danish study mainly focusing on junior hospital doctors did not find significantly higher statistics performance in physicians with previous biostatistics education or interest in research (10). These reports used different sets of questions compared to this study, thus hindering direct comparisons.

Approximately one out of five medical residents acknowledged below average familiarity with eight biostatistical concepts that are routinely encountered in the medical literature (12, 14). This lack of comfort was substantiated by the similar percentage of residents who answered all biostatistics questions erroneously. Residents considered themselves the least familiar with the concept of correlation coefficient. Indeed, their actual performance in the correlation coefficient quiz was clearly lower compared to all other tests. The independent association between self-reported biostatistics familiarity and knowledge score was further verified in a multivariate model (Table 4). It is of note that residents with advanced education through a master’s or PhD degree were significantly more likely to report higher familiarity with biostatistical concepts although this effect did not translate into substantial performance improvement on the knowledge questions. Previous education in biostatistics also tended to independently increase familiarity rankings although this association did not reach statistical significance. These observations indicate that although residents with advanced training and prior statistical education are more likely to believe that they are comfortable with statistics compared to other medical residents, they experience similar difficulties in interpreting research statistics. Consequently, formalized systematic teaching of biostatistics during residency will be required to ensure that residents are proficient in understanding and communicating statistical information.

To the best of our knowledge, the present study is the first to investigate the susceptibility of physicians to the gambler’s fallacy and the conjunction fallacy in medical settings. A recent survey in Germany reported that approximately 60% of the general population selected the correct answer in a coin toss gambler’s fallacy test (27). The pervasiveness of this bias among our study population of medical residents is alarming, especially given the fact that the bias was investigated using a problem within their domain of expertise. Residents who felt that the patient has increased chances of having tuberculosis are likely to overlook other important causes of hemoptysis. Notably, residents with higher biostatistics knowledge scores were less prone to the gambler’s fallacy. Therefore, continuing medical education programs focusing in statistical concepts and reasoning may mitigate the probability judgment biases of residents (34, 35). Furthermore, specific instruction aimed at increasing awareness of the gambler’s fallacy may reduce residents’ susceptibility to this bias (35–37).

Approximately 80–90% of respondents generally violate the conjunction rule according to previous reports using various presentations of the ‘Linda’ problem (29, 38, 39). In the present study, we found a slightly lower percentage (68.6%) of violations of class-inclusion in medical residents using a clinically based problem. This percentage was found to be independent of residents’ individual characteristics including age, gender, residency year, past biostatistics training, performance on the biostatistics tests and possession of other advanced degrees. A number of authors have argued that linguistic and pragmatic features in conjunction fallacy scenarios may lead respondents to alternative evaluations (38, 40). For example, if residents interpret the statement ‘John has CAD’ to additionally imply that ‘John does not have Huntington’s disease’ then ranking this statement as more probable than ‘John has CAD and Huntington’s disease’ would not be considered a conjunction fallacy. In addition, similarly to the English and German languages, the word ‘probable’ is polysemous in Greek and residents faced with multiple possible interpretations may infer a non-mathematical meaning of probability.

A further interpretation of the high number of conjunction violations found in the present study may be that residents apply different inferential rules and heuristic procedures resulting in answers that appear mathematically incorrect (41). Physicians use a repertoire of clinically relevant assumptions to arrive at a differential diagnosis based on the patient’s history, symptoms and physical examination. These diagnostic norms tend to focus on the most relevant information and assign that information its appropriate weight taking into account various clinical goals and expectations. This strategy may indeed disregard syntactical systems such as the conjunction rule. However, medical decision making is not dependent solely on the probability of an event (e.g., a disease). More often, clinicians focus on calculating the probability times payoff or cost when constructing a differential diagnosis list. Although this approach can lead physicians to rank the constituent as more probable than the conjunction it may also reflect an effective and intelligent way to tackle clinical uncertainty. During pretesting of our conjunction fallacy vignette, residents were specifically made aware of the mathematical nature of the test and the class-inclusion violation of ranking the probability of CAD and Huntington’s disease as more probably than Huntington’s disease. However, they still insisted that paying attention to the gist that the patient had multiple risk factors for CAD was far more reasonable than precise mathematical rankings.

### Limitations

The cross-sectional design of the present study prevented determination of causality. Furthermore, in order to fully protect residents’ anonymity, we were unable to collect any further data on non-respondents. Although the response rate was significantly higher than what is typical in surveys of physicians (42), we cannot exclude the possibility that residents who felt less comfortable with statistical concepts may have been less willing to complete and return the questionnaire. The present survey was purposely kept brief in order to achieve maximum participation. We thus limited the assessment of residents’ biostatistics knowledge to seven biostatistical concepts (Table 2) which however represent some of the most commonly used statistical methods found in the medical literature today.

## Conclusions

The measured biostatistics performance in our survey indicates that an alarmingly large number of medical residents are unable to correctly interpret crucial statistical concepts that are commonly found in the medical literature. These difficulties were pervasive, extending even to residents with prior biostatistics education and advanced graduate training. In addition, residents were found to be especially prone to the gambler’s fallacy bias, which may undermine clinical judgment and medical decision making. Frequent violations of the conjunction rule in a clinical scenario were also observed and residents were uncomfortable with the mathematical explanation of the problem. The low performance of medical residents in the biostatistics knowledge tests and the gambler’s fallacy question was independent of any prior biostatistics education, which mainly occurred during medical school. Therefore, in order to adequately develop and maintain the biostatistical reasoning of medical residents, educators may need to re-evaluate how such information is taught as well as emphasize and systematize the teaching of statistical concepts during residency.
